# Aberrant cerebrovascular reactivity presents as an early biomarker of psychosis susceptibility in patients with 22q11.2DS

**DOI:** 10.1038/s41398-026-04382-y

**Published:** 2026-08-14

**Authors:** Farnaz Delavari, Stefano Moia, Silas Forrer, Corrado Sandini, Nada Kojovic, Alessandro Pascucci, Clémence Lacour, Stephan Eliez, Dimitri Van De Ville

**Affiliations:** 1 Developmental Imaging and Psychopathology Laboratory, University of Geneva School of Medicine, Geneva, Switzerland; 2 Neuro-X Institute, École Polytechnique Fédérale de Lausanne, Geneva, Switzerland; 3 Radiology Department, Lausanne University Hospital (CHUV) and University of Lausanne (UNIL), Lausanne, Switzerland; 4 Fondation Pôle Autisme, Geneva, Switzerland; 5 Psychiatry Department, Faculty of Medicine, University of Geneva, Geneva, Switzerland; 6 Autism Brain and Behavior Lab, Faculty of Medicine, University of Geneva, Geneva, Switzerland; 7 Department of Genetic Medicine and Development, University of Geneva School of Medicine, Geneva, Switzerland; 8 Department of Radiology and Medical Informatics, University of Geneva (UNIGE), Geneva, Switzerland

**Keywords:** Neuroscience, Biomarkers

## Abstract

The brain’s ability to regulate blood flow is fundamental to both its function and development. In the context of neurodevelopmental disorders such as schizophrenia, understanding the complex interactions between cerebrovascular health and brain function is crucial for unraveling the pathophysiology of psychosis. This study investigates the developmental trajectory of cerebrovascular reactivity (CVR) in 22q11 deletion syndrome (22q11.2DS) compared to healthy controls, and its association with psychosis susceptibility. Using one of the largest longitudinal data-set of resting-state fMRI, we mapped for the first time voxel-level CVR across development. We found an early reduction in CVR in 22q11.2DS, and in particular in those who later developed positive psychotic symptoms (PPS + ). These reductions were evident within the anterior cingulate cortex, frontal lobes, and globi pallidi (GP). We propose that the pattern of CVR alterations presenting early during childhood is possibly linked to altered blood-brain barrier integrity or permeability, potentially involving endothelial or tight-junction dysfunction; however, BBB integrity was not directly measured in this study. A decrease in CVR during childhood and within the frontal regions and GP was predictive of subsequent development of positive psychotic symptoms), which often occurs during adolescence in 22q11.2DS patients. These findings suggest that cerebrovascular health is critical for normal brain development, particularly in regions like the striatum, which are vulnerable to vascular damage due to their anatomical features. These results underline the potential of CVR as an early biomarker for psychosis vulnerability, emphasizing the need for targeted interventions to mitigate neurodevelopmental disruptions of cerebrovascular health in 22q11.2DS.

## Introduction

Cerebrovascular reactivity (CVR), defined as the capacity of cerebral blood vessels to adjust their diameter in response to vasodilatory stimuli, such as increased CO2 concentration in the bloodstream, plays a pivotal role in maintaining gas homeostasis and cerebral perfusion and interacts with neurovascular coupling [1]. This complex physiological phenomenon, involving endothelial cells, pericytes, astrocytes, and neurons, plays a crucial role in maintaining brain function and responsiveness to metabolic demands [2, 3]. The coupling between vessels and neurons is mostly studied in the neuro-to-vascular direction. However, the vascular tone has also a direct impact on neuronal activity in the cortex [4]. Thus, the clinical implications of CVR are broad and impact various conditions of the brain. Indeed, CVR has been well studied in the domain of aging and cognitive function, where it has been shown that a decline in CVR predicts deterioration in cognitive functioning in older adults [5].

The implications of CVR abnormalities extend to neurodegenerative diseases, such as Alzheimer’s and Parkinson’s disease [6, 7], where decreased CVR is discussed to result in chronic hypoxia, giving rise to neuroinflammation and processes that contribute to disease progression. In the case of neuroinflammatory disorder, for instance multiple sclerosis (MS), chronic inflammation might lead to a reduced CVR, contributing to symptoms such as cognitive dysfunction and fatigue [8, 9]. Aside from global CVR reduction, regional variabilities in cerebrovascular reactivity, such as those observed particularly in the hippocampus, frontal, cingulate, and parietal areas, contribute to varied cognitive outcomes with age [10, 11]. These findings underscore the importance of spatial heterogeneity in vascular decline that may give rise to a unique set of neurological symptoms.

Studies investigating CVR in psychiatric disorders are scarce. Impaired perfusion has been associated with anxiety and other mood disorders[12]. Regions within the brainstem and amygdala expressing altered CVR have been implicated in mice models of anxiety disorders [13]. Moreover, two studies have documented reduced CVR in developmental psychiatric populations: notably among adolescents with bipolar disorder and young adults with depressive disorders, emphasizing on the importance of considering developmental hits to the cerebrovascular health in psychiatric disorders [14, 15]. Of note, to our knowledge there are no studies on CVR in children and adolescent populations in the context of prodromal phase of psychotic disorders, despite the evidence demonstrating that CVR measurements are reproducible in children. For instance, studies in children with risk of ischemia have demonstrated that CVR measurements can indicate tissue-level microvascular dysfunction [4, 16, 17], highlighting the utility of CVR assessments in pediatric studies. Indeed, it is imaginable for neurodevelopmental processes to rely heavily on cerebrovascular dynamics. CVR disruption during critical periods of brain development may lead to aberrant structural and functional alterations, consequently contributing to the emergence of psychopathological manifestations later in life [18]. Understanding how cerebrovascular dynamics affects abnormal brain development and psychopathology holds a significant promise for revealing underlying mechanisms and developing targeted interventions aimed at preventing neurodevelopmental disorders.

Schizophrenia, viewed primarily as a disorder characterized by onset of psychosis, is now increasingly recognized as a neurodevelopmental disorder with origins in early brain development [19]. This view highlights the importance of investigating the developmental trajectories preceding the onset of first symptoms of psychosis. However, characterizing brain maturation during the early premorbid stages of schizophrenia presents significant challenges, primarily due to the low incidence of psychosis in the general population [20]. To address this challenge, individuals with 22q11.2DS present a particularly valuable model [21]. With a 40% risk of progressing into psychosis, 22q11.2DS provides a unique opportunity to investigate the neurodevelopmental abnormalities that give rise to the onset of schizophrenia [22]. Additionally, 22q11.2DS represent a unique population for investigating the interplay between genetic predispositions, cerebrovascular dysfunction, and psychiatric manifestations.

Various mechanisms in 22q11.2DS may affect vascular health and thus cerebrovascular function, including congenital heart disease (CHD), endothelial dysfunction and vascular malformations that lead to an unstable vascular network, potentially impairing blood supply to the brain [23, 24]. Moreover, altered expression of genes involved in vascular and blood-brain barrier (BBB) integrity has been associated with presentation of psychosis in 22q11DS [25, 26]. Moreover, a study of mice-models with gene-deletion equivalent to 22q11.2DS, demonstrated early impairment of the BBB [27]. Furthermore, a recent preclinical study reported that neonatal oxytocin treatment in this mouse model was associated with improved BBB integrity [28]. These converging vulnerabilities suggest that cerebrovascular function measured by CVR may be altered early in 22q11.2DS and may contribute to atypical neurodevelopmental trajectories associated with psychosis susceptibility.

Performing CVR mapping on children is feasible [16] but challenging nonetheless. Indeed, gold standard approaches such as acetazolamide injection or gas challenges [1] are either not indicated or can cause discomfort in vulnerable subjects, such as children. Breathing challenges are normally employed as a more approachable and practical alternative, and while they are robust to certain lack of compliance [29], they are still too challenging for pediatric populations. However, a recent advancement in non-invasive functional magnetic resonance imaging (MRI) technique allows for the qualitative assessment of resting-state CVR (rCVR) [30], removing completely the constraint of subject compliance and providing an opportunity to employ this measurement in a developing human population to gain insights into neurovascular coupling and cerebrovascular health.

In this study, we leverage the large cohort of Geneva-22q11.2DS who underwent longitudinal resting state BOLD functional MRI, to measure rCVR, hereafter called CVR, in individuals with 22q11.2DS and healthy controls, aiming to characterize developmental changes in cerebrovascular function across the whole brain. Our investigation focuses on identifying if CVR impairments are present in patients with 22q11.2DS. Moreover, we aim to show when during the course of the development these impairments present themselves and whether they may contribute to the emergence of positive psychotic symptoms. Furthermore, we aim to investigate if part of these changes could be attributed to the congenital heart defects present in this population at birth. Given the strong genetic component on vascular system development and integrity [31, 32], individuals with 22q11.2DS possess inherent vulnerabilities in cerebrovascular health, which may play a role in their predisposition to a spectrum of neurodevelopmental abnormalities. We hypothesize that the markers of CVR impairment in critical brain regions are present at an early age already during childhood. We further hypothesize that cerebrovascular alteration are an early hit that are associated with later development of psychosis.

## Material and methods

### Participants

A total of 113 participants diagnosed with 22q11.2DS (49% female, age range 6-30 years) – with a confirmed genetic diagnosis of 22q11.2 deletion syndrome from a local genetics department, and 133 healthy control subjects (HCs) (56% female, age range 6–30 years) underwent multiple MRI scans. For the sample of HCs, we included participants from unaffected siblings of the patients or through an open call to the Geneva State School system in Switzerland. HCs were screened for history of any psychopathology and developmental abnormalities or cardiac anomalies and were matched for age and sex.

Deletion carriers underwent a comprehensive clinical assessment with an expert psychiatrist (SE) during each visit, during which Structured Interview for Prodromal Syndromes [33] (SIPS) was administered to assess the presence of attenuated symptoms of psychosis. One participant was too young (<6 years old) and was not assessed with SIPS. As studied in previous publications from the same cohort [34, 35], we used SIPS results to create subgroups based on the participants’ clinical characteristics with regards to psychosis vulnerability. Patients were classified as PPS(+) (n = 46) if they scored equal to or higher than 3 in at least one visit on any of the subscales for positive psychotic symptoms (Unusual Thought Content, Suspiciousness, Grandiosity, Hallucinations, and Disorganized Communication) [36]. The remaining participants were considered nonPPS. Participants’ clinical characteristics are further detailed in Table 1. IQ was assessed using age-appropriate Wechsler scales [37, 38]. Psychiatric diagnoses were established using standardized clinical interviews [39–41], and information on medication use and congenital heart defects was obtained from clinical records. Group comparisons reported in Table 1 were performed using independent-samples t-tests for continuous variables and chi-square tests for categorical variables.

**Table 1:** Table of demographics and participant characteristics.

	22q11.2DS	Healthy Control	P-value	PPS +	nonPPS	P-value
**Counts**	113	133	NAN	46	66	NAN
**Age** mean (sd)	18.401 (5.232)	17.540 (5.300)	0.099	18.592 (4.985)	18.342 (5.377)	0.739
**Sex** female %	49%	56%	0.158	54.8%	45.2%	0.185
**IQ** mean (sd)	71.420 (13.060)	111.393 (12.518)	<0.001	67.237 (11.302)	74.251 (13.582)	0.005 **
**White matter CVR** mean (sd)	0.029 (0.013)	0.027(0.014)	0.097	0.027 (0.013)	0.030 (0.013)	0.105
**Grey matter CVR** mean (sd)	0.059 (0.021)	0.060 (0.023)	0.515	0.055 (0.021)	0.061 (0.022)	0.058
**Scanner type** Scanner site (HUG/CB) %	68.5%	64.6%	0.404	73.8%	64.3%	0.158
**Congenital heart defect** female %	37 (54%)	NaN	NaN	17 (53%)	20 (55%)	0.9
**Anxiety disorder (%)**	54 (48%)	NaN	NaN	28 (61%)	26 (39%)	0.025 **
**Attention deficit hyperactivity disorder (%)**	56 (50%)	NaN	NaN	23 (50%)	32 (49%)	0.875
**Mood disorder (%)**	27 (24%)	NaN	NaN	15 (33%)	12 (18%)	0.079
**Schizophrenia spectrum disorders (%)**	11 (10%)	NaN	NaN	10 (22%)	0 (0%)	<0.001 **
**More than one psychiatric comorbidity (%)**	48 (56%)	NaN	NaN	26 (67%)	22 (49%)	0.001 **
**Second generation antipsychotic** (History of ever taking)	26 (31.3%) 30 unknown	NaN	NaN	21 (56.8%) 9 unknown	5 (10.9%) 20 unknown	<0.001 **
**Serotonin reuptake inhibitor** (History of ever taking)	73 (85.9%) 28 unknown	NaN	NaN	35 (92.1%) 8 unknown	38 (80.9%) 19 unknown	0.138

IQ measurement: Wechsler Intelligence Scale for Children-III for children [37] and Wechsler Adult Intelligence Scale-III for adults [38]. Presence of psychiatric disorders: Clinical interview with patients using the Diagnostic Interview for Children and Adolescents–Revised [39], the psychosis supplement from the Schedule for Affective Disorders and Schizophrenia for School-Age Children–Present and Lifetime version [40], and the Structured Clinical Interview for DSM-5 Axis I Disorders [41].NaN, not available; PPS(+), with positive psychotic symptoms; nonPPS, without positive psychotic symptoms; 22q11.2DS, 22q11DS deletion syndrome.

*HUG* geneva university hospitals, *CB* campus biotech.

* p < 0.05; ** p < 0.001.

Medication variables are reported at the participant level as history of ever taking the medication across available visits. Percentages were calculated among participants with available medication information; participants with unknown medication history are reported separately.

### Magnetic resonance imaging (MRI) acquisitions and parameters

The MR images were acquired on a 3 T Siemens MAGNETOM scanner (Siemens Healthineers, Erlangen, Germany). The structural images were obtained with a T1-weighted sequence comprising 192 slices, with a volumetric resolution of 0.86 × 0.86 × 1.1 mm³ (repetition time = 2500 ms, echo time = 3 ms, inversion time = 1100 ms, field of view = 23.5 cm², flip angle = 8°, acquisition matrix = 256 × 256, slice thickness = 1.1 mm, phase encoding right > left, no fat suppression). BOLD resting state fMRI scans, for duration of 8 min, were obtained using a T2*-weighted sequence (200 frames, acquisition matrix = 94 × 128, field of view = 96 × 128 mm², voxel size = 1.84 × 1.84 × 3.2 mm³, 38 axial slices, slice thickness = 3.2 mm, repetition time = 2400 ms, echo time = 30 ms, flip angle = 85°, phase encoding anterior > posterior, descending sequential ordering, GRAPPA acceleration mode with a factor for parallel imaging = 2). During resting-state scans, participants were instructed to fixate on a white cross on the screen, remain awake and let their mind wander [34].

A total of 448 scans were acquired and were first included in the pool of analysis. However, 34 scans were excluded during quality control due to excessive head motion, defined as translational displacement greater than 3 mm or rotational displacement greater than 3° during the scan. Four scans had a brain scan with the vertex of the brain out of the field of view and therefore were excluded. One scan was excluded because the subject fell asleep during the scan. Finally, 409 scans from 246 participants were retained for the calculation of CVR. The distribution of scans per participant and the timing of longitudinal follow-up are reported in the Supplementary Materials table.

### Preprocessing and cerebrovascular reactivity (CVR) map creation

The data analysis was carried out using Statistical Parametric Mapping (SPM) software (SPM12 Wellcome Trust Centre for Neuroimaging, London, UK; http://www.fil.ion.ucl.ac.uk/spm)) and custom MATLAB scripts (MathWorks, Natick, MA) which has been made publicly available at: https://github.com/farnazdelavari/fmri_preprocessing_mip. The preprocessing steps for the BOLD image series included motion correction, smoothing with a Gaussian filter at a full-width-half-maximum (FWHM) of 6 mm, and linear detrending. Following the methodology of Liu et al. we obtained a proxy of the pressure of arterial CO2 changes based on intrinsic BOLD fluctuations, by filtering the BOLD time series within a 0.02–0.04 Hz frequency band (Liu et al., 2017 and Pinto et al., 2020 [30, 42]). The average BOLD time course for this range was then computed considering a gray matter mask derived from a T1-weighted MPRAGE image, serving as the reference time course for gas fluctuations. Note that, because of the BOLD acquisition parameters, the cerebellum was removed from the field of view. Subsequently, a linear regression analysis was conducted using the reference time course as the independent variable and each voxel’s time course as the dependent variable. Motion parameters were also included as confounding factors, specifically the six rigid-body parameters (three translations and three rotations) estimated during realignment. Prior to model estimation, both the reference time course and voxel-wise BOLD time series (as well as motion regressors) were standardized (z-scored). This analysis generated a CVR index for each voxel, defined as the regression coefficient associated with the reference time course, resulting in a qualitative resting state CVR map for each participant at each visit. Because both predictors and voxel time series were normalized, the resulting CVR values are dimensionless standardized coefficients, reflecting the strength of coupling between local BOLD fluctuations and the global gray matter signal. Therefore, this measure represents a relative CVR proxy derived from resting-state fluctuations rather than an absolute physiological CVR (e.g., %BOLD/mmHg). Importantly, because the voxel-wise and reference time courses were z-scored before regression, this index is primarily driven by the temporal coupling between the reference gray-matter signal and each voxel-wise time course, rather than by the absolute magnitude of percent BOLD signal change. Therefore, two signals with similar temporal profiles but different absolute BOLD amplitudes may yield similar standardized CVR indices. These CVR maps were then warped into DARTEL space [43] and further into the Montreal Neurological Institute space [44] for groupwise analysis. To be noted that the use of standardized signals reduces the influence of global intensity inhomogeneities (e.g., receiver coil sensitivity profiles), although residual spatial bias cannot be entirely excluded.

### Mixed- effect model

To study the effect of age and clinical group (22q11DS vs HC) on global CVR, the average White Matter (WM) and Gray Matter (GM) CVR values for each subject were calculated using the WM and GM mask provided by the SPM segmentation separately. These values for the longitudinal sample were then used as dependent variables in two separate mixed-effect GLM models, one per tissue, with age, grouping and their interactions as independent variable, as well as sex as a covariate, using the publicly available toolbox (https://github.com/danizoeller/myMixedModelsTrajectories). Details of this analysis are described in the supplemental materials.

The results of these analysis are presented in detail in the supplemental materials (Figures S1, S5 and S6).

### Partial least squares - correlation

Partial least squares correlation (PLS-C) [45] was used to identify multivariate spatial patterns linked to group and developmental effects; permutation testing was used to assess component significance under the null distribution; and bootstrap ratios were used to assess the stability of voxel contributions to each latent component. In details, similar to prior published work of this group [46, 47], we added longitudinal age regressors on the behavior side of the myPLS toolbox, for which the implementation is publicly available (https://github.com/MIPLabCH/myPLS [48].

To briefly describe this analysis, the cross-covariance matrix (R) is computed between a set of design variables (Y) and the CVR maps per subject at each visit (X). The design side (Y) consists of variables encoding the binary variable of diagnosis (or psychosis susceptibility grouping); as well as the longitudinal age regressors and their interaction. These longitudinal age regressors are described as follows: The “mean_age” that encodes for each visit of the subject the average age of that subject across all visits. The “delta_age” assigns the difference between the chronological age of a subject at the time of the scan and the subject’s mean age across all visits. Of note, by design these two age regressors are orthogonalized and can be interpreted as the cross-sectional and longitudinal effect of age, respectively. We next devise additional regressors to calculate the interaction of group and age regressors to enable the model to show deviation of the developmental trajectories of one group with respect to the other. The covariance matrix (R = Y^T^X) is then computed and undergoes singular value decomposition (R = USV^T^) resulting in latent components (LC)s. These LCs are tested for significance by permutation testing (1000 permutations). The component was considered significant if the p-value was less than 0.01. This threshold was determined by multiple comparisons correction using the Bonferroni method, dividing the usual significance level of 0.05 by the number of components tested [5], resulting in a corrected significance level of 0.01. Details of the PLS-C are outlined in the supplemental materials and the null distribution of the singular values are depicted in supplemental figure S7. To check the stability of the results and account for the possibility of overfitting, we obtained bootstrap ratios obtained by performing bootstrapping with replacement (500 bootstrap samples). Bootstrap ratio scores were thresholded at an absolute value greater than 3, which corresponds to a 99% confidence interval not crossing zero and indicates a stable contribution from this variable to the LC [45].

Additional PLS-C analyses were conducted to assess the potential influence of congenital heart disease (CHD), antipsychotic medication use, and IQ. These variables were used as grouping or covariate factors in separate models following the same procedure as described above.

## Results

### Sample characteristics and global tissue-level CVR

A total of 409 longitudinal scans from 246 participants were included in the analysis. The sample comprised 113 participants with 22q11.2DS, including 46 individuals classified as PPS + , and 133 healthy controls. Demographic and clinical characteristics are summarized in Table 1, together with statistical tests assessing potential group differences.

We first examined whether CVR alterations were detectable at the global tissue level by comparing average gray-matter and white-matter CVR between groups and by testing age-by-group interaction effects. This analysis was intended to determine whether 22q11.2DS or psychosis vulnerability was associated with a broad, whole-tissue alteration in cerebrovascular responsiveness. We then proceeded to spatially resolved voxel-wise analyses because CVR alterations may be regionally heterogeneous and age-dependent, and could therefore be missed when averaged across the whole gray or white matter.

No significant group or age-by-group interaction effect was detected for average gray-matter or white-matter CVR when comparing participants with 22q11.2DS to healthy controls, nor when comparing 22q11.2DS participants with and without psychosis vulnerability. These results, presented in Supplemental Figure S1, indicate that CVR alterations were not expressed as global tissue-level differences, supporting the use of regional multivariate analyses in the following sections.

### 22q11.2DS shows an early offset in regional cerebrovascular reactivity trajectories

**Fig. 1:**
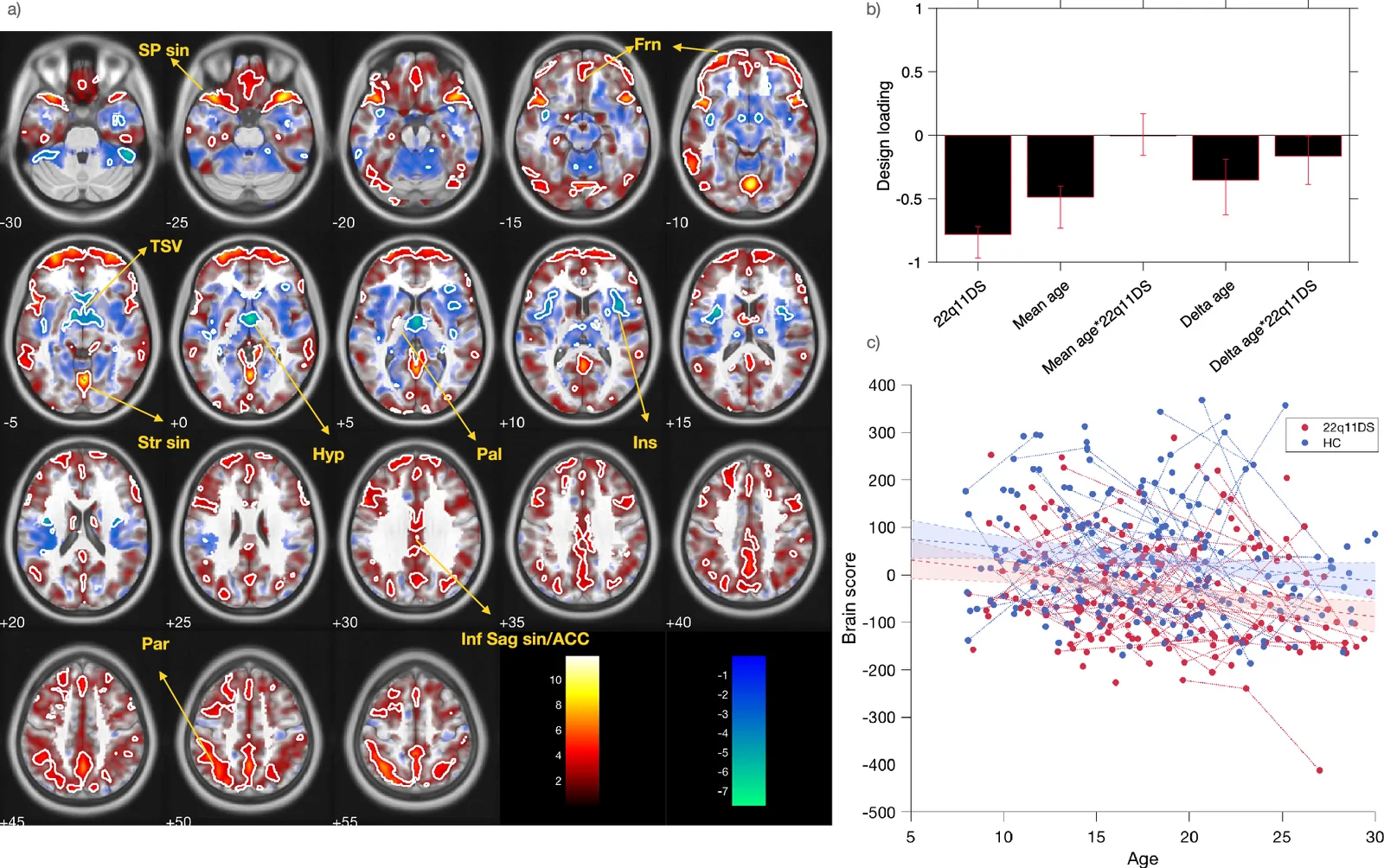
Multivariate voxel-wise pattern of CVR trajectories in 22q11.2DS versus HCs. Panel **a**) presents the spatial results of the component for which the colors indicate the effect size of the contribution each voxel to the component. The transparency of each voxel indicates the statistical strength of its contribution to the component. Clusters for which the strength is indicated statistically stable (as indicated by a bootstrap ratio with an absolute value greater than 3) are contoured with the white line– SP sin: Sphenoparietal Sinus; Str sin: Straight Sinus; Inf Sag sin: inferior sagittal sinus; ACC: Anterior cingulate cortex; Frn: Frontal; Par: parietal; Ins : Insula; Hyp : Hypothalamus; TSV : Thalamostriate veins; Pal : Globus Pallidus (GP). **b**) Bar plot summarizing loadings on designs variables included in the PLS-corrlation in relation to the pattern depicted in Panel a. Error bars represent 95% confidence interval. Bars depicted in black represented the design loadings with a stable contribution across bootstraps c) Scatter plot showing the relationship between age and the brain score for individuals with 22q11.2 deletion syndrome and healthy controls. Fitted regression lines show the age-related trend within each group, with shaded bands indicating 95% confidence.

The longitudinal voxel-wise CVR analysis comparing the age trajectories in deletion carriers and HC, using PLS-C, resulted in one significant component (p-value = 0.002) (Fig. 1).

This component captured a steady age-effect as indicated by the stable age loadings as well as a prominent group-effect as indicated by the stable loading on 22q11.2DS diagnosis (Fig. 1b). As illustrated in Fig. 1c, the group-age relationship is indicative of linear age relationship in both groups with a significant early offset in deletion carriers. Figure 1a demonstrates the multivariate brain pattern associated with this age-diagnosis changes. Two sets of regions are revealed by this PLS-C. A first set of regions displayed in red demonstrate a steady decrease in CVR with lower values in participants with 22q11.2DS. This decrease is mainly observed within the major vasculature of the brain such as Sphenoparietal, Straight, and Inferior Sagittal sinuses as well as anterior cingulate cortex (ACC). Similarly, regions within the frontal and parietal lobe demonstrated a decreased CVR within the 22q11.2DS group. Conversely, within the Hypothalamus, Insula, and Thalamostriate veins, which are demonstrated in blue, CVR values increased with age while deletion carriers showed higher CVR values with regards to their HC counterparts. It is worth noting that both set of regions are revealed within a multivariate analysis. As such, participants with 22q11.2DS who demonstrate a decrease of CVR in red regions concurrently demonstrate an increase of CVR in blue regions.

Overall, this component suggests that 22q11.2DS is associated with an early shift in regional CVR trajectories, combining reduced CVR in frontal, cingulate, parietal, and venous regions with relatively increased CVR in hypothalamic, insular, and thalamostriate regions.

### Positive psychotic symptoms are associated with earlier cerebrovascular reactivity reductions in psychosis-relevant regions

**Fig. 2:**
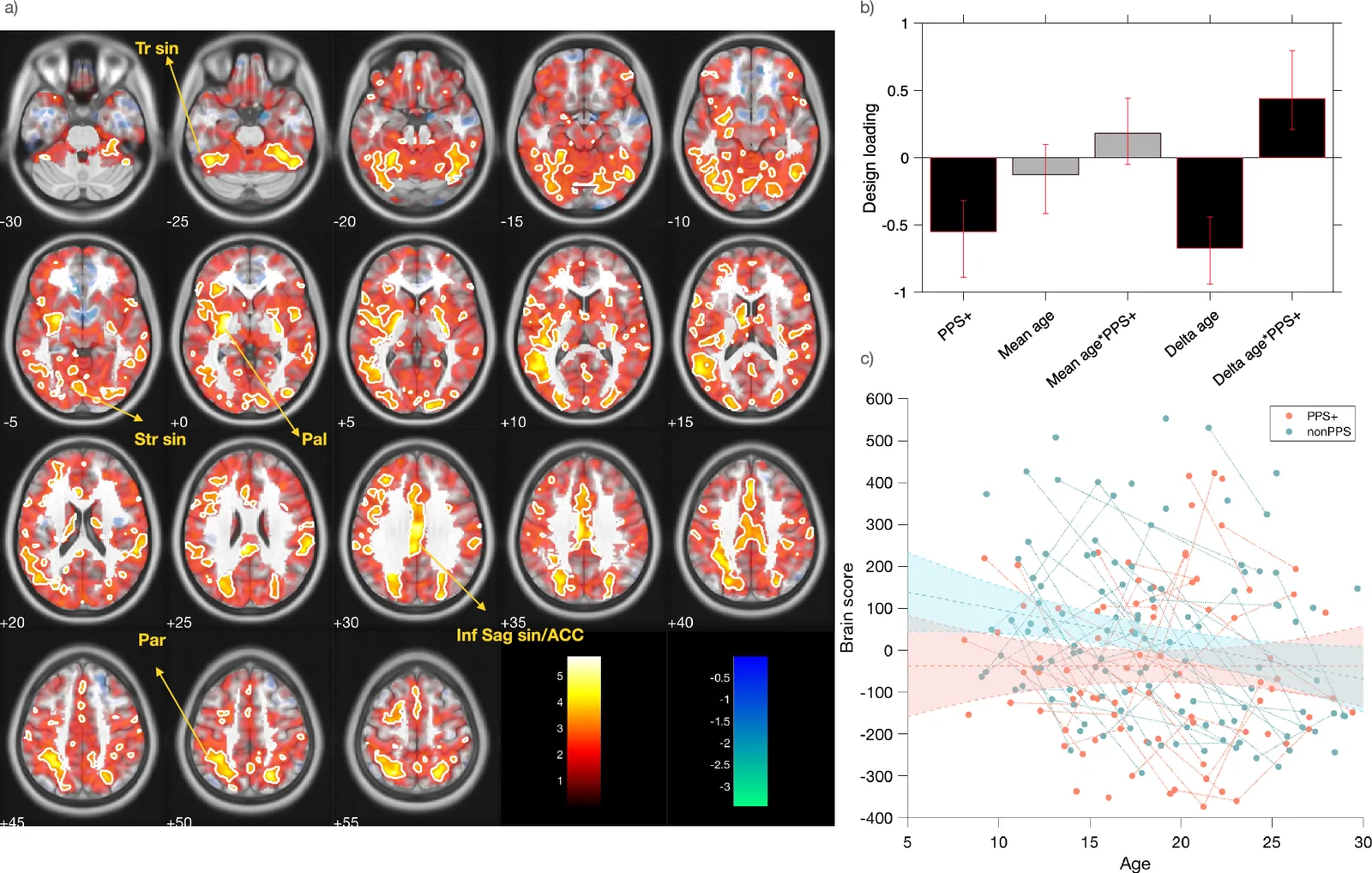
Multivariate voxel-wise pattern of CVR trajectories in PPS+ versus nonPPS. Panel **a**) presents the spatial results of the component for which the colors indicate the effect size of the contribution each voxel to the component. The transparency of each voxel indicates the statistical strength of its contribution to the component. Clusters for which the strength is indicated statistically stable (as indicated by a bootstrap ratio with an absolute value greater than 3) are contoured with the white line. _Str sin: Straight Sinus; Inf Sag sin : inferior sagittal sinus; ACC: Anterior cingulate cortex; Par: parietal; Tr sin : Transverse Sinus; Pal : Globus Pallidus (GP). **b**) Bar plot summarizing loadings on designs variables included in the PLS-corrlation in relation to the pattern depicted in Panel a. Error bars represent 95% confidence interval. Bars depicted in black represented the design loadings with a stable contribution across bootstraps **c**) Scatter plot showing the relationship between age and the brain score for individuals with 22q11.2 deletion syndrome and healthy controls. Fitted regression lines show the age-related trend within each group, with shaded bands indicating 95% confidence.

The PLS-C comparing the age relationship between PPS+ and nonPPS group revealed one significant component (p-value = 0.002) (Fig. 2).

This component revealed a multivariate brain pattern mainly within Inferior Sagittal sinus, ACC Transverse sinus, Straight sinus, GP, and clusters within the parietal lobe (Fig. 2a). These clusters showed a prominent negative loading on the PPS+ group (Fig. 2b) as well as a positive age-interaction loading. The PLS-C analysis revealed a significant effect of delta-age and its interaction with group, indicating an early decrease of CVR within the above-mentioned regions in PPS+ group, whereas the nonPPS group reached this level of low CVR later in the aging process. Figure 2c illustrates this pattern.

Finally, in an exploratory classification analysis performed across predefined age windows, the PPS-related CVR brain score best distinguished PPS+ from nonPPS participants during childhood and early adolescence. The highest cross-validated classification performance was observed in the 8:14-year age window, suggesting that the CVR pattern associated with later positive psychotic symptoms is already most detectable early in development (Supplementary Table S2).

These findings suggest that the CVR pattern associated with later positive psychotic symptoms is already detectable during childhood and early adolescence, before the typical age of psychosis onset.

### The observed cerebrovascular reactivity patterns are not explained by congenital heart disease, IQ, or antipsychotic medication

We performed 3 complementary analyses to investigate the effect of CHD in CVR, as well as two possible confounding variables namely antipsychotic medication intake and IQ which are significantly different across PPS+ and nonPPS groups. None of these PLS-Cs resulted in a component reaching the significance level. Nonetheless, we visualize and discuss the first component of each of these PLS-C in the supplementary materials.

These complementary analyses suggest that the main CVR patterns are more closely related to 22q11.2DS diagnosis and psychosis vulnerability than to congenital heart disease, IQ, or antipsychotic medication exposure.

## Discussion

To the best of our knowledge, this is the first study to examine CVR and in-vivo markers of cerebrovascular health in patients with 22q11.2DS. We conducted a multivariate analysis on a longitudinal cohort of 22q11.2DS to explore whole-brain CVR and its developmental alterations across deletion carriers, focusing particularly on a subpopulation with increased susceptibility to psychosis. This study provides initial evidence of an early hit to cerebrovascular health and its role in the pathophysiology of psychosis.

### Cereberovascular reactivity alterations during development

When investigating global WM and GM cerebrovascular reactivity, no age-related changes were detected. However, a voxel-wise assessment revealed anatomical regions where CVR varied with age. Little evidence is at hand regarding CVR alterations during development, with the majority of studies focusing on older populations [5, 6, 9–11, 29, 30]. However, our results align with studies on the effects of aging on CVR, showing that while global WM and GM CVR remain stable over time, regional variability may be present across different brain areas in relation to age [11]. Additionally, it has been demonstrated that the rate of vascular aging may differ across various brain regions, with one study showing that the temporal lobe experiences the fastest rate of longitudinal CVR decline in middle-aged individuals, followed by the parietal and frontal lobes[5]. Our results demonstrate a steady decrease in CVR within frontal, parietal and striatal regions during development during adolescence and young adulthood. As expected, the majority of clusters in the multivariate pattern showed a decrease of CVR with age. Regional decreases in CVR during aging is well documented [5, 9–11], and has been associated with normal loss of vascular wall elasticity and environmental factors such as inflammation. The decrease we observed during childhood and adolescence may as well be associated with the normal loss of elasticity in vasculature that already starts during childhood in central and peripheral blood vessels [49]. Remarkably, the multivariate nature of this study allowed for detection of a set of regions with a relative increase in CVR over age. This observation may be related to “steal” phenomena [50], where lower CVR of certain regions flushes the blood volume into other regions with more CVR capacity. Such areas might be initially sub-performing compared to other regions [51]. Alternatively, a reduction in CVR might be related to the development of a different hemodynamic delay in CVR [52], especially for regions characterized by bigger vessels. An interesting case is the increase of CVR over age in the hypothalamus. The hypothalamic paradoxical change in BOLD-derived CVR is reminiscent of studies demonstrating the inverse neurovascular coupling in the hypothalamus area due to neurons secreting vasopressin [53]. It is possible that hypothalamus demonstrates a contradictory CVR response of vasoconstriction to accommodate the specialized function of the region, similar to evidence in literature in nuclei within brainstem specialized for breathing, that paradoxically show vasoconstriction upon increase of CO2 [13] to signal respiratory stimulation.

### Linking cerebrovascular health to psychosis susceptibility

Overall, our multivariate results showed that during childhood CVR is higher in clusters within the frontal and parietal lobe and major brain sinuses, and paradoxically lower within the hypothalamus and insular regions. Moreover, this normative CVR profile would become less present as participants grow. In addition to the age effect, we demonstrate that participants with 22q11.2DS show an early offset with regards to their CVR development, and are more similar to older HC individuals than their chronological age. Indeed, participants with 22q11.2DS may exhibit a defect in their CVR profile due to several interrelated factors. Individuals with 22q11.2DS are more susceptible to immunosuppression and thus multiple infections [54]. Moreover, metabolic disturbances such as obesity are more prevalent in the 22q11.2DS population [55]. The chronic state of inflammation raised by obesity or multiple infections may eventually accelerate vascular aging and compromise CVR as suggested by the literature [56–58].

Nevertheless, while findings indicate an early offset in CVR in deletion carriers, this pattern does not aggravate over time. Therefore, the causes presented at birth may be more plausible to contribute to this pattern. Among these causes, congenital factors inherent to 22q11.2DS, such as cardiovascular abnormalities and vascular anomalies are pivotal. Contrary to our initial hypothesis we did not find any alterations of the CVR in patients who had a history of CHD and those who did not. However, independent from the presence of CHD [24], the deletion carriers are haploinsufficient for the claudin-5 gene (CLDN5) which leads to endothelial tight junction dysfunction and disruption of blood-brain barrier integrity which may contribute to the compromised CVR in 22q11.2DS population [26]. Indeed, BBB impairment has been directly linked to CVR decline in animal models. In a recent study in rat brains, when the BBB was locally opened using focused ultrasound, [59] attenuated CVR response, measured by the CO2 gas challenge, occurred in the affected region. Therefore, our results regarding CVR reduction in 22q11.2DS may be in part linked to the established BBB impairment in this population.

**Fig. 3:**
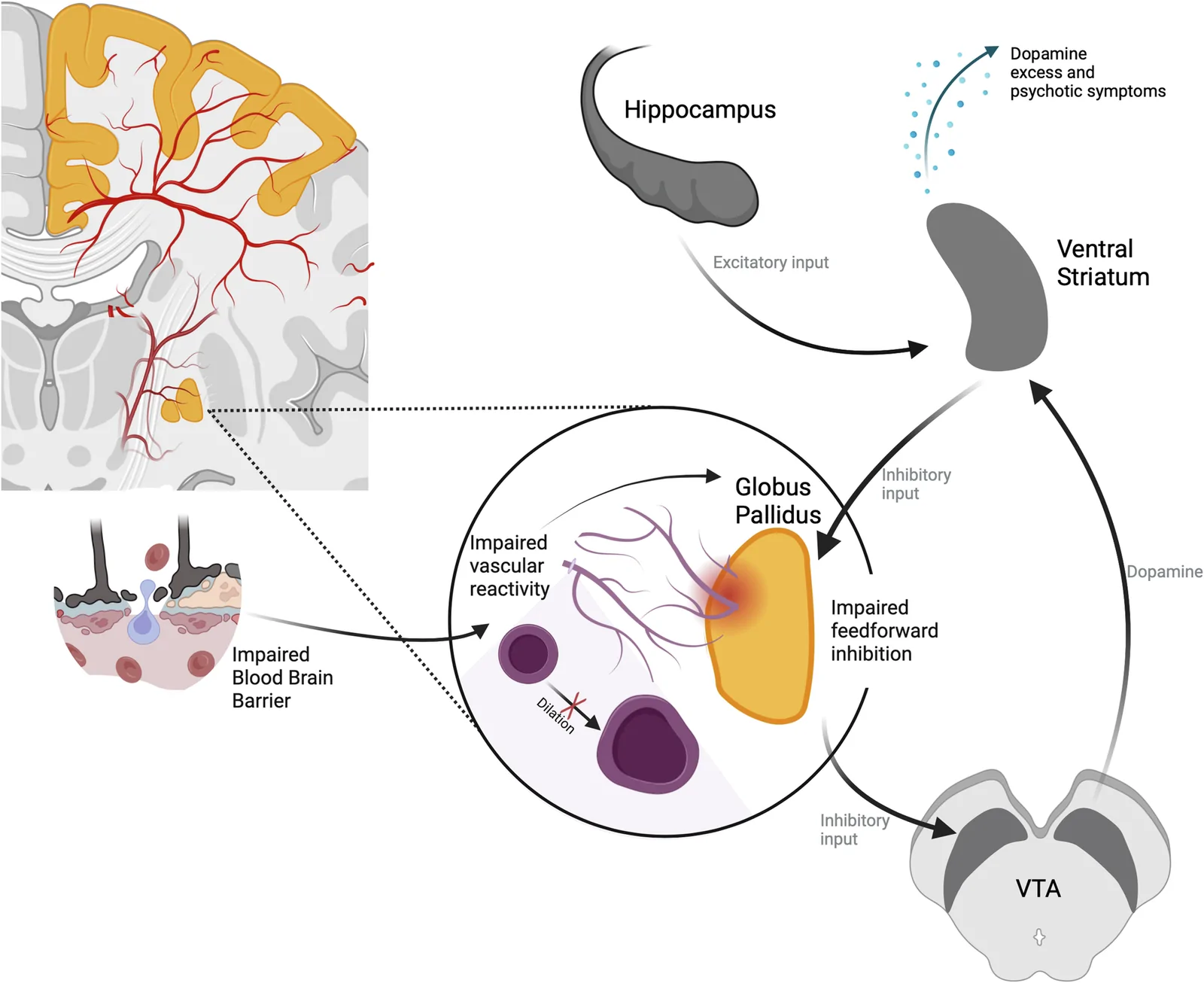
Putative mechanism through which cerebrovascular reactivity may increase the risk for development of psychosis. Altered cerebrovascular reactivity may represent blood-brain-barrier impairment, which could permit the entry of peripheral inflammatory or neuroactive substances and disrupt metabolic homeostasis, thereby contributing to abnormal development and functional impairment in vulnerable susceptible brain regions. Among those, the globus pallidus plays a key role in the feed-forward inhibition of sub-cortical dopaminergic signaling. Disruption of this inhibitory control may lead to disinhibition of dopaminergic neurons, particularly within the ventral tegmental area (VTA), and altered hippocampal regulation of dopamine signaling. Such dopaminergic dysregulation is a central mechanism implicated in the pathophysiology of psychosis. This proposed pathway remains hypothetical and is not directly tested in the present study.

Moreover, deletion carriers who eventually developed mild to moderate PPS+ demonstrated an early decrease in CVR within the ACC, frontal lobe, and the GP. In contrast, the group of patients without such susceptibility to psychosis (nonPPS) only exhibited this level of decreased CVR as a result of aging, by adulthood. This early reduction in CVR in these critical regions might thus play a crucial role in the later development of psychosis. Notably, the significant role of the ACC and frontal regions in schizophrenia, as well as in 22q11.2DS, has been well documented [60, 61] in relation to cognitive control, salience monitoring, and affective regulation. Similarly, parietal alterations may reflect broader disruption of attentional and associative networks [62]. On the other hand, alterations involving the hypothalamus and insula may point to broader autonomic, homeostatic, or interoceptive processes [53, 63], whereas findings involving large venous structures should be interpreted more cautiously as reflecting hemodynamic or vascular BOLD-related components rather than isolated regional neuronal dysfunction [64]. Additionally, the GP plays a key role in a critical circuit involved in psychosis, which includes the hippocampus, ventral striatum, and ventral tegmental area (VTA) (Fig. 3). Briefly, increased excitatory inputs from the hippocampus to the ventral striatum decreases the inhibition of the ventral pallidum, resulting in heightened activity of VTA neurons projecting to the striatum [65]. This results in a downstream hyperdopaminergic state, which is believed to be a major underlying mechanism of psychotic symptoms [66].

Of note, the majority of deletion carriers first express psychotic symptoms during early adolescence [22]. However, our results revealed the pattern of decreased CVR to be present already at childhood. Therefore, it is possible that early dysmaturation of the GP, due to abnormal CVR, may lead to a more pronounced lack of feedforward inhibition, making the population more susceptible to eventual hippocampal hyperactivity and the downstream hyperdopaminergic state that is documented during adolescent in this population [46, 67]. Therefore, the cerebrovascular health of the striatum may present an additional factor as an “early hit” that plays a role in the complex pathobiology of psychosis. This finding aligns well with the literature discussing the specific susceptibility of striatum vasculature due to particular anatomical features of this region. The striatum is served by the lenticulostriate arteries with narrow diameters, rendering them more vulnerable to vascular damage. This is significant as over 40% of strokes stem from severe blockages within the basal ganglia’s vessels [68, 69]. Additionally, hippocampus and the striatum are shown to be more susceptible to BBB impairment within the cortex [49], with recent evidence showing BBB impairment within the hippocampus is more transient, but persists within the striatum. Notably, a recent study [70] showed that lower CVR within the striatum was associated with markers of BBB impairment. Overall, this evidence suggests the critical role of the vascular health and BBB integrity, especially within the striatum, for normal development. In light of these findings, our findings regarding a decreased CVR in young individuals with 22q11.2DS could remark the early BBB impairment in these individuals.

### Methodological considerations and future perspectives

The results presented in this study must be interpreted within the context of several methodological considerations.

First, here CVR was estimated from resting-state BOLD recordings rather than measured using a gas challenge or direct physiological recordings. Therefore, the present maps should be interpreted as qualitative resting-state relative CVR indices rather than quantitative physiological CVR measurements. Although resting-state relative CVR has been proposed and validated against CO₂-based CVR measures, this validation remains limited. In particular, the original validation was conducted in a relatively small sample of healthy adults [30]. Moreover, our analysis used the 0.02–0.04 Hz band-pass filter proposed in the original Liu et al.[30] approach, whereas later implementations of rs-CVR computation recommend a broader frequency range. Thus, although our approach enables the investigation of CVR-like vascular fluctuations in a large longitudinal pediatric and clinical cohort where gas challenges are difficult to implement, and that this method has been verified and utilized in several studies [42], the resulting findings should be interpreted cautiously as relative and method-dependent markers of cerebrovascular function. Future studies incorporating simultaneous physiological CO₂ recordings or gas-challenge CVR would be required to validate the regional specificity of these effects in 22q11.2DS.

Second, disentangling the effect of medication intake and PPS+ presents a significant challenge as inevitably, the majority of the PPS+ group have a history of taking antipsychotic medications. In order to better understand the effects of medication, we performed a secondary analysis using history of taking second generation antipsychotics as the grouping contrast. This PLS-C did not lead to a component reaching the significance level. Albeit the pattern of the first component in this non-significant PLS-C was similar to the one of the PPS+ vs nonPPS PLS-C (Fig S3). However, as antipsychotic medication is shown to improve blood flow in the striatum [71], it is unexpected that the reduced CVR is derived by the medication intake. A more detailed investigation regarding the effect of medication is available in the Supplementary materials. Future studies with information regarding onset of medication intake as well as duration and dosage of medication are needed to further delineate the role of medication intake in CVR.

Additionally, increased CVR in 22q11.2DS within certain brain regions was contrary to our initial hypothesis. Indeed, the multivariate nature of our analysis is not allowing for deducting conclusions on individual regions and results should be considered as a pattern. Therefore, the increase of CVR within regions of the brain happens concurrent to the decrease of CVR to other regions. Future studies incorporating a lagged-CVR methodology may be helpful to further elucidate these results and whether this observation could be attributed to the steal phenomena.

Finally, although BBB dysfunction is a biologically plausible mechanism in 22q11.2DS and may be relevant to the CVR alterations observed here, our study did not include any direct measure of BBB integrity or permeability. Therefore, the relationship between CVR alterations and BBB dysfunction cannot be established from the present data and should be considered hypothesis-generating rather than demonstrated. Future multimodal studies combining CVR with direct measures of BBB integrity will be necessary to test this proposed link.

In short, we showed a substantially early decrease of CVR values in a group of deletion carriers with eventual development of positive psychotic symptoms. We demonstrate this decrease is present during childhood (approximately 8-12 years), several years before the typical onset of symptoms in adolescence (approximately 15-20 years) [72]; and is most prominent within the GP, a key region for inhibiting dopaminergic cascades. Hence, we provide the first evidence linking cerebrovascular health in childhood to psychosis susceptibility in 22q11.2DS. Based on the trajectories of the alterations, decreased CVR may present an early biomarker of psychosis susceptibility. We propose a model in which cerebrovascular integrity must be considered among the first hits in the pathophysiology of psychosis, possibly through early damage to the striatum caused by neuroinflammation and mediated by BBB impairment. Further research should be conducted to disentangle the possible relationship of BBB impairment and CVR within the critical brain regions in young individuals with 22q11.2DS. These studies could elaborate whether this biomarker is also a potential treatment target for restoring BBB function as a preventive measure for psychosis.

## Supplementary information

Supplementary information

## Data Availability

Due to ethical and data protection restrictions, the datasets of current study are not publicly available. However, anonymized data supporting the findings of this study would be available from the corresponding author upon reasonable request and subject to appropriate institutional and ethical approvals.

## Code availability

The custom MATLAB scripts used for preprocessing and resting-state CVR map generation are publicly available at: https://github.com/farnazdelavari/fmri_preprocessing_mip. The PLS-C analyses were performed using the publicly available myPLS toolbox: https://github.com/MIPLabCH/myPLS.

## References

[1] Fisher JA, Mikulis DJ. Cerebrovascular reactivity: purpose, optimizing methods, and limitations to interpretation - a personal 20-year odyssey of (Re)searching. Front Physiol. 2021;12:629651. 10.3389/fphys.2021.629651 PMC8047146 33868001

[2] Hall CN, Reynell C, Gesslein B, Hamilton NB, Mishra A, Sutherland BA, et al. Capillary pericytes regulate cerebral blood flow in health and disease. Nature. 2014;508:55–60. 10.1038/nature13165 PMC3976267 24670647

[3] Howarth C, Sutherland BA, Choi HB, Martin C, Lind BL, Khennouf L, et al. A critical role for astrocytes in hypercapnic vasodilation in brain. Journal of Neuroscience. 2017;37:2403–14. 10.1523/JNEUROSCI.0005-16.2016 PMC5354350 28137973

[4] Kim KJ, Ramiro Diaz J, Iddings JA, Filosa JA. Vasculo-neuronal coupling: retrograde vascular communication to brain neurons. J Neurosci. 2016;36:12624–39. 10.1523/JNEUROSCI.1300-16.2016 PMC5157107 27821575

[5] Peng SL, Chen X, Li Y, Rodrigue KM, Park DC, Lu H. Age-related changes in cerebrovascular reactivity and their relationship to cognition: A four-year longitudinal study. Neuroimage. 2018;174:257–62. 10.1016/j.neuroimage.2018.03.033 PMC5949266 29567504

[6] Ryman SG, Shaff N, Dodd A, Nitschke S, Wertz C, Julio K, et al. Reduced and delayed cerebrovascular reactivity in patients with Parkinson’s disease. Mov Disord. 2023;38:1262–72. 10.1002/mds.29429 PMC10524339 37157056

[7] Sur S, Lin Z, Li Y, Yasar S, Rosenberg P, Moghekar A, et al. Association of cerebrovascular reactivity and Alzheimer pathologic markers with cognitive performance. Neurology. 2020;95:e962–72. 10.1212/WNL.0000000000010133 PMC7668551 32661101

[8] D’Haeseleer M, Hostenbach S, Peeters I, Sankari SE, Nagels G, De Keyser J, et al. Cerebral hypoperfusion: a new pathophysiologic concept in multiple sclerosis?. J Cereb Blood Flow Metab. 2015;35:1406–10. 10.1038/jcbfm.2015.131 PMC4640326 26104292

[9] Pelizzari L, Laganà MM, Baglio F, Bergsland N, Cecconi P, Viotti S, et al. Cerebrovascular reactivity and its correlation with age in patients with multiple sclerosis. Brain Imaging Behav. 2020;14:1889–98. 10.1007/s11682-019-00132-5 31175576

[10] Catchlove SJ, Parrish TB, Chen Y, Macpherson H, Hughes ME, Pipingas A. Regional cerebrovascular reactivity and cognitive performance in healthy aging. J Exp Neurosci. 2018;12:1179069518785151. 10.1177/1179069518785151 PMC6043917 30013388

[11] McKetton L, Sobczyk O, Duffin J, Poublanc J, Sam K, Crawley AP, et al. The aging brain and cerebrovascular reactivity. Neuroimage. 2018;181:132–41. 10.1016/j.neuroimage.2018.07.007 29981482

[12] Harandi AA, Kimia N, Medghalchi A, Sharifipour E, Pakdaman H, Siavoshi F, et al. Cerebral hemodynamic response to generalized anxiety disorder. Psychiatry Research: Neuroimaging. 2023;333:111654. 10.1016/j.pscychresns.2023.111654 37229961

[13] Wenzel J, Hansen CE, Bettoni C, Vogt MA, Lembrich B, Natsagdorj R, et al. Impaired endothelium-mediated cerebrovascular reactivity promotes anxiety and respiration disorders in mice. Proceedings of the National Academy of Sciences. 2020;117:1753–61. 10.1073/pnas.1907467117 PMC6983400 31896584

[14] Urback AL, Metcalfe AW, Korczak DJ, MacIntosh BJ, Goldstein BI. Reduced cerebrovascular reactivity among adolescents with bipolar disorder. Bipolar Disord. 2019;21:124–31. 10.1111/bdi.12719 30422372

[15] Darling AM, Richey RE, Akins JD, Saunders EFH, Matthew Brothers R, Greaney JL. Cerebrovascular reactivity is blunted in young adults with major depressive disorder: The influence of current depressive symptomology. J Affect Disord. 2021;295:513–21. 10.1016/j.jad.2021.08.061 PMC8667006 34509066

[16] Leung J, Kim JA, Kassner A. Reproducibility of cerebrovascular reactivity measures in children using BOLD MRI. J Magn Reson Imaging. 2016;43:1191–5. 10.1002/jmri.25063 26435493

[17] Dlamini N, Yau I, Westmacott R, Shroff M, Armstrong D, Logan W, et al. Cerebrovascular reactivity and intellectual outcome in childhood stroke with transient cerebral arteriopathy. Pediatr Neurol. 2017;69:71–8. 10.1016/j.pediatrneurol.2017.01.001 28258787

[18] Kim JA, Leung J, Lerch JP, Kassner A. Reduced cerebrovascular reserve is regionally associated with cortical thickness reductions in children with sickle cell disease. Brain Res. 2016;1642:263–9. 10.1016/j.brainres.2016.03.041 27026656

[19] Insel TR. Rethinking schizophrenia. Nature. 2010;468:187–93. 10.1038/nature09552 21068826

[20] Charlson FJ, Ferrari AJ, Santomauro DF, Diminic S, Stockings E, Scott JG, et al. Global epidemiology and burden of schizophrenia: findings from the global burden of disease study 2016. Schizophr Bull. 2018;44:1195–203. 10.1093/schbul/sby058 PMC6192504 29762765

[21] Gur RE, Roalf DR, Alexander-Bloch A, McDonald-McGinn DM, Gur RC. Pathways to understanding psychosis through rare – 22q11.2DS - and common variants. Current Opinion in Genetics & Development. 2021;68:35–40. 10.1016/j.gde.2021.01.007 PMC8728946 33571729

[22] Schneider M, Debbané M, Bassett AS, Chow EWC, Fung WLA, van den Bree MBM, et al. Psychiatric disorders from childhood to adulthood in 22q11.2 deletion syndrome: results from the international consortium on brain and behavior in 22q11.2 deletion syndrome. American Journal of Psychiatry. 2014;171:627–39. 10.1176/appi.ajp.2013.13070864 PMC4285461 24577245

[23] Yi JJ, Tang SX, McDonald-McGinn DM, Calkins ME, Whinna DA, Souders MC, et al. Contribution of congenital heart disease to neuropsychiatric outcome in school-age children with 22q11.2 deletion syndrome. Am J Med Genet B Neuropsychiatr Genet. 2014;165b:137–47. 10.1002/ajmg.b.32215 PMC4154196 24265253

[24] Unolt M, Versacci P, Anaclerio S, Lambiase C, Calcagni G, Trezzi M, et al. Congenital heart diseases and cardiovascular abnormalities in 22q11.2 deletion syndrome: from well-established knowledge to new frontiers. Am J Med Genet A. 2018;176:2087–98. 10.1002/ajmg.a.38662 PMC6497171 29663641

[25] Greene C, Kealy J, Humphries MM, Gong Y, Hou J, Hudson N, et al. Dose-dependent expression of claudin-5 is a modifying factor in schizophrenia. Mol Psychiatry. 2018;23:2156–66. 10.1038/mp.2017.156 PMC6298981 28993710

[26] Crockett AM, Kebir H, Anderson SA, Jyonouchi S, Romberg N, Alvarez JI. 22q11.2 deletion-associated blood-brain barrier permeability potentiates systemic capillary leak syndrome neurologic features. J Clin Immunol. 2024;44:87. 10.1007/s10875-024-01686-w PMC11490314 38578402

[27] Crockett AM, Ryan SK, Vásquez AH, Canning C, Kanyuch N, Kebir H, et al. Disruption of the blood-brain barrier in 22q11.2 deletion syndrome. Brain. 2021;144:1351–60. 10.1093/brain/awab055 PMC8219368 33876226

[28] Castellani G, Ciampoli M, Benedetti A, Ferretti V, Trigilio G, Barcik W, et al. Oxytocin seals the blood–brain barrier, improving 22q11.2 deletion syndrome trajectories. Brain. 2025;148:3184–98. 10.1093/brain/awaf112 40662347

[29] Solis-Barquero SM, Echeverria-Chasco R, Calvo-Imirizaldu M, Cacho-Asenjo E, Martinez-Simon A, Vidorreta M, et al. Breath-hold induced cerebrovascular reactivity measurements using optimized pseudocontinuous arterial spin labeling. Front Physiol. 2021;12:621720. 10.3389/fphys.2021.621720 PMC7925895 33679436

[30] Liu P, Li Y, Pinho M, Park DC, Welch BG, Lu H. Cerebrovascular reactivity mapping without gas challenges. Neuroimage. 2017;146:320–6. 10.1016/j.neuroimage.2016.11.054 PMC5321860 27888058

[31] Cioffi S, Martucciello S, Fulcoli FG, Bilio M, Ferrentino R, Nusco E, et al. Tbx1 regulates brain vascularization. Hum Mol Genet. 2014;23:78–89. 10.1093/hmg/ddt400 23945394

[32] Greene C, Hanley N, Campbell M. Claudin-5: gatekeeper of neurological function. Fluids Barriers CNS. 2019;16:3. 10.1186/s12987-019-0123-z PMC6350359 30691500

[33] Miller TJ, McGlashan TH, Rosen JL, Cadenhead K, Ventura J, McFarlane W, et al. Prodromal assessment with the structured interview for prodromal syndromes and the scale of prodromal symptoms: predictive validity, interrater reliability, and training to reliability. Schizophr Bull. 2003;29:703–15. 10.1093/oxfordjournals.schbul.a007040 14989408

[34] Zöller D, Padula MC, Sandini C, Schneider M, Scariati E, Van De Ville D, et al. Psychotic symptoms influence the development of anterior cingulate BOLD variability in 22q11. 2 deletion syndrome. Schizophr Res. 2018;193:319–28. 10.1016/j.schres.2017.08.003 28803847

[35] Scariati E, Schaer M, Richiardi J, Schneider M, Debbané M, Van De Ville D, et al. Identifying 22q11. 2 deletion syndrome and psychosis using resting-state connectivity patterns. Brain Topogr. 2014;27:808–21. 10.1007/s10548-014-0356-8 24562717

[36] Fusar-Poli P, Borgwardt S, Bechdolf A, Addington J, Riecher-Rössler A, Schultze-Lutter F, et al. The psychosis high-risk state: a comprehensive state-of-the-art review. JAMA Psychiatry. 2013;70:107–20. 10.1001/jamapsychiatry.2013.269 PMC4356506 23165428

[37] Wechsler D. Wechsler Intelligence Scale for Children: Manual. New York, NY: Psychological Corporation. 1949.

[38] Wechsler D. Manual for the Wechsler Adult Intelligence Scale. New York, NY: Psychological Corporation. 1955.

[39] Reich W. Diagnostic interview for children and adolescents (DICA). Journal of the American Academy of Child & Adolescent Psychiatry. 2000;39:59–66. 10.1097/00004583-200001000-00017 10638068

[40] Kaufman J, Birmaher B, Brent D, Rao U, Flynn C, Moreci P, et al. Schedule for affective disorders and schizophrenia for school-age children-present and lifetime version (K-SADS-PL): initial reliability and validity data. Journal of the American Academy of Child & Adolescent Psychiatry. 1997;36:980–8. 10.1097/00004583-199707000-00021 9204677

[41] First MB, Spitzer RL, Gibbon M, Williams JBW. Structured Clinical Interview for DSM-IV Axis I Disorders (SCID-I), Clinician Version, Administration Booklet. Washington, DC: American Psychiatric Press. 1997.

[42] Pinto J, Bright MG, Bulte DP, Figueiredo P. Cerebrovascular reactivity mapping without gas challenges: a methodological guide. Front Physiol. 2020;11:608475. 10.3389/fphys.2020.608475 PMC7848198 33536935

[43] Ashburner J. A fast diffeomorphic image registration algorithm. Neuroimage. 2007;38:95–113. 10.1016/j.neuroimage.2007.07.007 17761438

[44] Collins DL, Zijdenbos AP, Kollokian V, Sled JG, Kabani NJ, Holmes CJ, et al. Design and construction of a realistic digital brain phantom. IEEE Trans Med Imaging. 1998;17:463–8. 10.1109/42.712135 9735909

[45] Krishnan A, Williams LJ, McIntosh AR, Abdi H. Partial Least Squares (PLS) methods for neuroimaging: a tutorial and review. Neuroimage. 2011;56:455–75. 10.1016/j.neuroimage.2010.07.034 20656037

[46] Delavari F, Sandini C, Zöller D, Mancini V, Bortolin K, Schneider M, et al. Dysmaturation observed as altered hippocampal functional connectivity at rest is associated with the emergence of positive psychotic symptoms in patients with 22q11 deletion syndrome. Biol Psychiatry. 2021;90:58–68. 10.1016/j.biopsych.2020.12.033 33771350

[47] Forrer S, Delavari F, Sandini C, Rafi H, Preti MG, Van De Ville D, et al. Longitudinal analysis of brain function-structure dependencies in 22q11.2DS and psychotic symptoms. Biological Psychiatry: Cognitive Neuroscience and Neuroimaging. 2024;9:882–95. 10.1016/j.bpsc.2024.05.008 38849032

[48] McIntosh AR, Lobaugh NJ. Partial least squares analysis of neuroimaging data: applications and advances. Neuroimage. 2004;23:S250–S63. 10.1016/j.neuroimage.2004.07.020 15501095

[49] Senzaki H, Akagi M, Hishi T, Ishizawa A, Yanagisawa M, Masutani S, et al. Age-associated changes in arterial elastic properties in children. Eur J Pediatr. 2002;161:547–51. 10.1007/s00431-002-1025-6 12297902

[50] Conklin J, Fierstra J, Crawley AP, Han JS, Poublanc J, Mandell DM, et al. Impaired cerebrovascular reactivity with steal phenomenon is associated with increased diffusion in white matter of patients with Moyamoya disease. Stroke. 2010;41:1610–6. 10.1161/STROKEAHA.110.579540 20576954

[51] Arteaga DF, Strother MK, Faraco CC, Jordan LC, Ladner TR, Dethrage LM, et al. The vascular steal phenomenon is an incomplete contributor to negative cerebrovascular reactivity in patients with symptomatic intracranial stenosis. J Cereb Blood Flow Metab. 2014;34:1453–62. 10.1038/jcbfm.2014.106 PMC4158662 24917040

[52] Stickland RC, Zvolanek KM, Moia S, Caballero-Gaudes C, Bright MG. Lag-optimized blood oxygenation level dependent cerebrovascular reactivity estimates derived from breathing task data have a stronger relationship with baseline cerebral blood flow. Front Neurosci. 2022;16:910025. 10.3389/fnins.2022.910025 PMC9254683 35801183

[53] Roy RK, Althammer F, Seymour AJ, Du W, Biancardi VC, Hamm JP, et al. Inverse neurovascular coupling contributes to positive feedback excitation of vasopressin neurons during a systemic homeostatic challenge. Cell Rep. 2021;37:109925. 10.1016/j.celrep.2021.109925 PMC9488983 34731601

[54] Jawad AF, McDonald-McGinn DM, Zackai E, Sullivan KE. Immunologic features of chromosome 22q11.2 deletion syndrome (DiGeorge syndrome/velocardiofacial syndrome). J Pediatr. 2001;139:715–23. 10.1067/mpd.2001.118534 11713452

[55] Voll SL, Boot E, Butcher NJ, Cooper S, Heung T, Chow EW, et al. Obesity in adults with 22q11.2 deletion syndrome. Genet Med. 2017;19:204–8. 10.1038/gim.2016.98 PMC5292049 27537705

[56] Frosch OH, Yau PL, Osorio RS, Rusinek H, Storey P, Convit A. Insulin resistance among obese middle-aged is associated with decreased cerebrovascular reactivity. Neurology. 2017;89:249–55. 10.1212/WNL.0000000000004110 PMC5513815 28615420

[57] Spencer JI, Bell JS, DeLuca GC. Vascular pathology in multiple sclerosis: reframing pathogenesis around the blood-brain barrier. Journal of Neurology, Neurosurgery & Psychiatry. 2018;89:42–52. 10.1136/jnnp-2017-316011 28860328

[58] Chiarelli AM, Villani A, Mascali D, Petsas N, Biondetti E, Caporale A, et al. Cerebrovascular reactivity in multiple sclerosis is restored with reduced inflammation during immunomodulation. Sci Rep. 2022;12:15453. 10.1038/s41598-022-19113-8 PMC9474533 36104366

[59] Todd N, Zhang Y, Livingstone M, Borsook D, McDannold N. The neurovascular response is attenuated by focused ultrasound-mediated disruption of the blood-brain barrier. Neuroimage. 2019;201:116010. 10.1016/j.neuroimage.2019.116010 PMC6765430 31302253

[60] Mattiaccio LM, Coman IL, Thompson CA, Fremont WP, Antshel KM, Kates WR. Frontal dysconnectivity in 22q11.2 deletion syndrome: an atlas-based functional connectivity analysis. Behav Brain Funct. 2018;14:2. 10.1186/s12993-018-0134-y PMC5775582 29352808

[61] Padula MC, Scariati E, Schaer M, Eliez S. A mini review on the contribution of the anterior cingulate cortex in the risk of Psychosis in 22q11.2 deletion syndrome. Front Psychiatry. 2018;9:372. 10.3389/fpsyt.2018.00372 PMC6107828 30174623

[62] Zhao B, Wang Y, Wang Y, Ji C, Zhang N. Changes in regional cerebral blood flow associated with psychosis in patients with Alzheimer’s disease. Int Psychogeriatr. 2026;38:100190. 10.1016/j.inpsyc.2026.100190 41633856

[63] Craig AD. How do you feel? Interoception: the sense of the physiological condition of the body. Nat Rev Neurosci. 2002;3:655–66. 10.1038/nrn894 12154366

[64] Olman CA, Inati S, Heeger DJ. The effect of large veins on spatial localization with GE BOLD at 3 T: displacement, not blurring. Neuroimage. 2007;34:1126–35. 10.1016/j.neuroimage.2006.08.045 17157534

[65] Egerton A, Grace AA, Stone J, Bossong MG, Sand M, McGuire P. Glutamate in schizophrenia: Neurodevelopmental perspectives and drug development. Schizophr Res. 2020;223:59–70. 10.1016/j.schres.2020.09.013 33071070

[66] McCutcheon RA, Abi-Dargham A, Howes OD. Schizophrenia, dopamine and the striatum: from biology to symptoms. Trends Neurosci. 2019;42:205–20. 10.1016/j.tins.2018.12.004 PMC6401206 30621912

[67] Mancini V, Saleh MG, Delavari F, Bagautdinova J, Eliez S. Excitatory/inhibitory imbalance underlies hippocampal atrophy in individuals with 22q11.2 deletion syndrome with psychotic symptoms. Biol Psychiatry. 2023;94:569–79. 10.1016/j.biopsych.2023.03.021 37011759

[68] Feekes JA, Cassell MD. The vascular supply of the functional compartments of the human striatum. Brain. 2006;129:2189–201. 10.1093/brain/awl158 16815876

[69] Park J. Movement disorders following cerebrovascular lesion in the basal ganglia circuit. J Mov Disord. 2016;9:71. 10.14802/jmd.16005 PMC4886205 27240808

[70] Zachariou V, Pappas C, Bauer CE, Shao X, Liu P, Lu H, et al. Regional differences in the link between water exchange rate across the blood–brain barrier and cognitive performance in normal aging. GeroScience. 2024;46:265–82. 10.1007/s11357-023-00930-2 PMC10828276 37713089

[71] Goozée R, Handley R, Kempton MJ, Dazzan P. A systematic review and meta-analysis of the effects of antipsychotic medications on regional cerebral blood flow (rCBF) in schizophrenia: association with response to treatment. Neuroscience & Biobehavioral Reviews. 2014;43:118–36. 10.1016/j.neubiorev.2014.03.014 24690578

[72] Schneider M, Schaer M, Mutlu AK, Menghetti S, Glaser B, Debbané M, et al. Clinical and cognitive risk factors for psychotic symptoms in 22q11.2 deletion syndrome: a transversal and longitudinal approach. Eur Child Adolesc Psychiatry. 2014;23:425–36. 10.1007/s00787-013-0469-8 PMC4042012 23999732

