# Supplementary material for: Aberrant cerebrovascular reactivity presents as an early biomarker of psychosis susceptibility in patients with 22q11.2DS
Source: Transl Psychiatry. 2026 Aug 14;16:630. doi: 10.1038/s41398-026-04382-y (PMC13752332; doi:10.1038/s41398-026-04382-y)
Supplement: Supplementary file 1 [file 41398_2026_4382_MOESM1_ESM.docx]

**Aberrant cerebrovascular reactivity presents as an early biomarker of psychosis susceptibility in patients with 22q11.2DS**

**Distribution and timing of longitudinal scans**

The final analytic sample included 409 scans from 246 participants: 209 scans from 133 healthy controls and 200 scans from 113 participants with 22q11.2DS. The age range at scanning was 7.96–29.98 years in healthy controls and 8.12–29.67 years in participants with 22q11.2DS.

Across the full sample, 127 participants had one scan, 79 had two scans, 36 had three scans, and 4 had four scans. In the healthy control group, 75 participants had one scan, 40 had two scans, and 18 had three scans. In the 22q11.2DS group, 52 participants had one scan, 39 had two scans, 18 had three scans, and 4 had four scans.

Among participants with repeated scans, the interval between the first and last available scan had a mean of 5.66 years and a median of 4.95 years, ranging from 0 to 12.21 years.

**Global gray- and white-matter CVR remains stable across age**

Average CVR of grey matter and white matter voxels were compared across groups over the age-span. Herewith tissue-level or voxel level average rs-rCVR trajectories were tested while accounting for repeated measurements of each subject:

$$CVR_{s}={\beta_{0}+\beta}_{1}group_{s}+\beta_{2}age_{s}+\beta_{3}\left( age_{s}*group_{s} \right)+\beta_{4}sex_{s}+\Theta_{s}$$

CVR_s_ is the average WM, GM or voxel CVR value of subject *s*, group_s_ is the group of subject s, age_s_ is the age of subject s, sex_s_ is the sex of subject s, and $\Theta$_s_ is the random effect of the *sth* subject. Mixed-effects trajectory models included an explicit fixed intercept and a subject-level random effect to account for repeated observations. Group was entered as the binary grouping variable required by the toolbox, with healthy controls/nonPPS coded as 0 and 22q11.2DS/PPS+ coded as 1. This coding was chosen because these were the primary contrasts of interest. Age was mean-centered prior to model estimation to facilitate interpretation of main effects, particularly the group factor. Sex was initially encoded as a binary variable (1 = female and 0 = male) and entered as a covariate; it was mean-centered internally by the toolbox before model estimation. The fitted design matrix included group, centered-age, age-by-group interaction, and centered sex-covariate terms. Therefore, the group coefficient represents the difference between group 1 and group 0 adjusted for centered sex, rather than a comparison restricted to male controls. Group and age-by-group effects were assessed using likelihood-ratio comparisons between the full model and reduced models excluding the corresponding terms.

*Figure S1- Comparison of global gray matter (GM) and white matter (WM) trajectories in groups. The significance of the effect of group (22q11 vs. HC or PPS+ vs. nonPPS) is demonstrated as “p-val group effect”. The significance of the effect of group (22q11 vs. HC or PPS+ vs. nonPPS) and age interaction is demonstrated as “p-value interaction “. Sex was included in all four models as a covariate. The group-level trajectory (solid line) is derived from mixed-effect model). The colored bands indicate the 95% confidence interval around the estimated group-level trajectory. Subjects’ repeated time-points are connected by dashed lines.*

**Results of the post-hoc analysis**

**CHD**

The PLS-C comparing the difference of CVR development in groups of deletion carriers with and without CHD resulted in first component with p-value=0.014.

Figure S2

However, as depicted in Figure S2, this component captured mainly the effect of aging and the CHD grouping had minimal effect with bootstrap-ratios crossing 0 (Figure S1-b).

**IQ**

A similar component (p-value=0.011) with major age pattern was observed when comparing deletion carriers with IQ higher or lower than median IQ = 70. As depicted in figure S3, this PLS also showed minimal and unstable loading on IQ grouping (Figure S3-b).

Figure S3

**Antipsychotics intake**
The PLS-C comparing deletion carriers who ever took second generation antipsychotic medication (Medication+) with those who did not (nonMedication), resulted in a first component with a p-value = 0.013.

Figure S4

This component was very similar to the result of the PLS-C for PPS+ and nonPPS. These results are expected as inherently, there is substantial overlap between patients who take the medication and the PPS+ patients (Refer to Table-1 in the main manuscript). However, the group effect of the medication intake is smaller than the one of the PPS+.

Additionally, we performed a partial correlation analysis. We calculated the partial correlation of the brain-scores of the component demonstrating reduction in CVR, with both variables of PPS+/nonPPS and Medication+/ nonmedication. This analysis showed no significant partial correlation of the brain-score and the medication intake.

Table S1

| Partial correlation with brain-score | Partial correlation co-efficient | P-value |
| --- | --- | --- |
| History of taking second generation | 0.0582 | 0.42 |
| PPS+ | 0.1616 | 0.023* |

We believe these results suggest the effect of belonging to the PPS+ group has a stronger contribution to the brain pattern observed in the PLS-C when compared to medication intake. Therefore, the results observed on medication intake may be explained by the overlap of the populations who are PPS+ and have a positive history for medication.

**Exploratory voxel-wise univariate analysis and cluster-based visualization**

To address a traditional univariate assessment, we performed complementary exploratory voxel-wise linear mixed-effects analyses. These analyses were conducted in addition to the primary multivariate PLS-C analysis and were intended to provide more direct regional interpretability of group-, age-, and interaction-related CVR effects.

For each in-mask voxel, CVR was modeled using a linear mixed-effects model with age, group, and the age-by-group interaction as fixed effects. Participant ID was included as a random intercept to account for repeated observations.

The model was specified as:

$$CVR_{s}=\beta_{1}group_{s}+\beta_{2}age_{s}+\beta_{3}\left( age_{s}*group_{s} \right)+\Theta_{s}$$

Where group was either diagnosis or the status for the PPS.

At each voxel, beta coefficients and p-values were extracted for the main effect of group, the main effect of age, and the age-by-group interaction. Figure S5 and S6 demonstrate voxels with signifcant p-values (uncorrected p≤0.05) for the age, group and age-by-group interaction effect in each model.

Figure S5 Exploratory voxel-wise univariate maps for the diganosis analysis.

Voxel-wise linear mixed-effects models were used to assess age, diangonsis status, and age-by-diagnosis interaction effects on CVR. Maps are thresholded at nominal uncorrected p≤0.05. Plots show mean CVR extracted within selected clusters and are provided for visualization of effect direction only.

Figure S6 . Exploratory voxel-wise univariate maps for the PPS analysis.

Voxel-wise linear mixed-effects models were used to assess age, PPS status, and age-by-PPS interaction effects on CVR. Maps are thresholded at nominal uncorrected p≤0.05. Plots show mean CVR extracted within selected clusters and are provided for visualization of effect direction only.

In these voxel-wise maps no FDR multiple-comparison correction was applied therefore, these voxel-wise results were interpreted as exploratory and descriptive rather than as confirmatory whole-brain statistical evidence.

To facilitate interpretation of the voxel-wise findings, within significant clusters, mean CVR was extracted for each participant/scan. These values were then plotted as a function of age and group to illustrate the trajectory of the said area.

Overall, the exploratory voxel-wise univariate analyses demonstrated similar results to the PLS-C findings by showing that selected components of the multivariate CVR patterns could also be visualized using traditional univariate models. For example, impaired CVR in the globuls pallidus of PPS+ and PPS- deletion carriers showed a similar trajectory as discussed in the main results of the PLS-C analysis. However, we note because the voxel-wise maps were not corrected for multiple comparisons and because the areas were derived from the same exploratory clusters, these analyses should be interpreted as descriptive support for the primary multivariate findings rather than as independent confirmatory evidence.

**Exploratory age-windowed prediction of PPS status from the CVR brain score**

To further evaluate whether the predictive value of CVR varied across development, we performed an exploratory age-windowed classification analysis within the 22q11.2DS group. This analysis was designed to identify the developmental age range in which the PPS-related CVR brain score most strongly discriminated deletion carriers with positive psychotic symptoms (PPS+) from those without positive psychotic symptoms (nonPPS).

For each scan, we used the brain score derived from the PPS-related PLS-C component as the predictor variable. Participants were divided into four non-overlapping age windows: 8–14, 14–20, 20–26, and 26–32 years. Within each age bin, only the one scan per participant was retained to avoid repeated-measure leakage; when multiple scans from the same participant were available within a bin, the scan closest to the center of the age bin was selected.

Within each age bin, PPS status was classified using logistic regression with the CVR brain score as the only predictor. Predictive performance was evaluated using stratified cross-validation. Model performance was summarized using the area under the receiver operating characteristic curve (ROC-AUC), balanced accuracy, sensitivity for PPS+ classification, and specificity for nonPPS classification. Confidence intervals for ROC-AUC were estimated using bootstrap resampling, and permutation testing was used to assess whether classification performance exceeded chance level.

***Results :***

Table S2

| **Age bin** | **Number** | **nonPPS** | **PPS+** | **ROC-AUC** | **95% CI** | **Permutation p-value** | **Balanced accuracy** | **Sensitivity** | **Specificity** |
| --- | --- | --- | --- | --- | --- | --- | --- | --- | --- |
| 8–14 | 39 | 24 | 15 | 0.71 | 0.51–0.86 | 0.015 | 0.66 | 0.73 | 0.58 |
| 14–20 | 69 | 38 | 31 | 0.66 | 0.52–0.78 | 0.019 | 0.63 | 0.65 | 0.61 |
| 20–26 | 48 | 26 | 22 | 0.39 | 0.23–0.56 | 0.897 | 0.42 | 0.41 | 0.42 |
| 26–32 | 18 | 11 | 7 | 0.52 | 0.22–0.83 | 0.474 | 0.58 | 0.43 | 0.73 |

The strongest discrimination between PPS+ and nonPPS participants was observed in the 8–14-year age window. In this bin, the CVR brain score classified PPS status with ROC-AUC = 0.71, 95% CI = 0.51–0.86, permutation p = 0.015, balanced accuracy = 0.66, sensitivity = 0.73, and specificity = 0.58. Classification performance was weaker in later age bins, suggesting that the discriminative value of the PPS-related CVR brain score is strongest during childhood and early adolescence.

These findings provide additional support for the interpretation that CVR alterations may represent an early marker of psychosis susceptibility in 22q11.2DS. However, because the brain score was derived from the PPS-related PLS-C component, this analysis should be interpreted as exploratory age-windowed discrimination rather than independent external validation.

**Details of included variables in PLS-C**

In the PLS-C design variables, we begin by incorporating a binary diagnostic variable. Depending on the analysis, this variable separates either HCs from patients with 22q11DS or PPS(+) from non-PPS individuals. Considering the study's longitudinal nature, we devised age-related variables as outlined below to capture both overall cohort aging effects and individual age-related changes.

Firstly, we use mean-age, defined as the average age of each subject across all study visits. Secondly, delta-age represents the difference between a subject's age at a specific visit and their mean-age. As detailed in our previous publication, the orthogonalization of mean-age and delta-age ensures they correspond to cross-sectional and longitudinal aging effects, respectively.

The matrix U contains a left singular vector for each latent component, representing the saliences of seven design variables. These saliences indicate how each design variable contributes to the brain-design correlation. The diagonal matrix S holds the singular values for each latent component, quantifying the extent to which each component explains the correlation. The matrix V, containing the right singular vectors, represents the brain saliences, which express the contribution of each voxel to the correlation captured by the component.

**Permutation testing and null distribution of singular values**

In the PLS-C analyses, permutation testing was used to evaluate the statistical significance of each latent component. This procedure was not used as a voxel-wise multiple-comparison correction method. Instead, it tested whether the singular value of a given latent component was larger than expected under the null hypothesis of no systematic association between the CVR maps and the design variables.

For each PLS-C analysis, the rows of the design matrix were randomly permuted relative to the brain matrix across 1000 permutations. At each permutation, the cross-covariance matrix between the permuted design matrix and the CVR data was recomputed, singular value decomposition was repeated, and the singular value of the latent component was stored. This generated an empirical null distribution of singular values for each component.

The observed singular value was then compared with this null distribution. The permutation-derived p-value was defined as the proportion of permuted singular values that were equal to or greater than the observed singular value. Components were considered significant if they survived the Bonferroni-corrected threshold of p < 0.01, corresponding to correction across the five tested latent components.

Figure S7 shows the permutation-derived null distributions for the first latent component in the two primary PLS-C analyses. In both cases, the observed singular value was located in the extreme right tail of the null distribution, supporting the statistical significance of the component.

Figure S7 : The permutation-derived null distributions for the first latent component in the two primary PLS-C analyses. S7-a : Comparing 22q11DS vs Healthy Controls (HC) and S7-b: Comparing PPS+ group vs PPS-. In both cases, the observed singular value was supporting the statistical significance of the component.
